# RootAnalyzer: A Cross-Section Image Analysis Tool for Automated Characterization of Root Cells and Tissues

**DOI:** 10.1371/journal.pone.0137655

**Published:** 2015-09-23

**Authors:** Joshua Chopin, Hamid Laga, Chun Yuan Huang, Sigrid Heuer, Stanley J. Miklavcic

**Affiliations:** 1 Phenomics and Bioinformatics Research Centre, University of South Australia, Mawson Lakes, South Australia, Australia; 2 The Australian Centre for Plant Functional Genomics, Urrbrae, South Australia, Australia; University of Nottingham, UNITED KINGDOM

## Abstract

The morphology of plant root anatomical features is a key factor in effective water and nutrient uptake. Existing techniques for phenotyping root anatomical traits are often based on manual or semi-automatic segmentation and annotation of microscopic images of root cross sections. In this article, we propose a fully automated tool, hereinafter referred to as *RootAnalyzer*, for efficiently extracting and analyzing anatomical traits from root-cross section images. Using a range of image processing techniques such as local thresholding and nearest neighbor identification, RootAnalyzer segments the plant root from the image’s background, classifies and characterizes the cortex, stele, endodermis and epidermis, and subsequently produces statistics about the morphological properties of the root cells and tissues. We use RootAnalyzer to analyze 15 images of wheat plants and one maize plant image and evaluate its performance against manually-obtained ground truth data. The comparison shows that RootAnalyzer can fully characterize most root tissue regions with over 90% accuracy.

## Introduction

Cereals are of global importance due to their use as staple food crops, renewable energy sources and livestock feed. As such, understanding the endogenous processes within cereal plants in order to increase their yield and tolerance to stress has become a priority for the plant biology community. However, the sheer volume of data that is required to engender a more significantly yielding plant variety is a bottleneck for the biologist who must often resort to manual and time consuming experiments and analysis. The focus of such experiments can be broadly split into two interrelated categories; plant shoot and plant root. The high-throughput analysis of plant shoot traits, growth and development is an actively researched field where, by using image processing and analysis, data can be acquired and subsequently analysed in a non-destructive manner [1–8]. In the case of plant roots, focus can be split further into two areas; root architecture, which is responsible for anchorage and uptake and foraging of water and nutrients, and root anatomy, which is important for the internal transport of water and nutrients. Root system architecture characterization has received a considerable amount of attention through the use of both destructive and nondestructive phenotyping technologies. For instance, root system architecture can be studied in a non-destructive manner by either imaging plants grown in soils using X-ray CT scanning devices, or by using standard RGB sensors when the plants are grown in transparent media. With the introduction of these methods has come a steady increase in focus on the 3D modeling of root architecture systems [9–12]. In contrast, methods for studying the anatomy of plant roots are less prevalent [13]. This is despite the fact that root anatomy plays a pivotal role in physiological function governing the overall growth of plants in a number of important ways [14–16] including the initiation of lateral [17, 18] and branch [19] roots, nutrient uptake [20, 21], stress tolerance [22–24] and tolerance to mechanical impedance [25].

**Fig 1:**
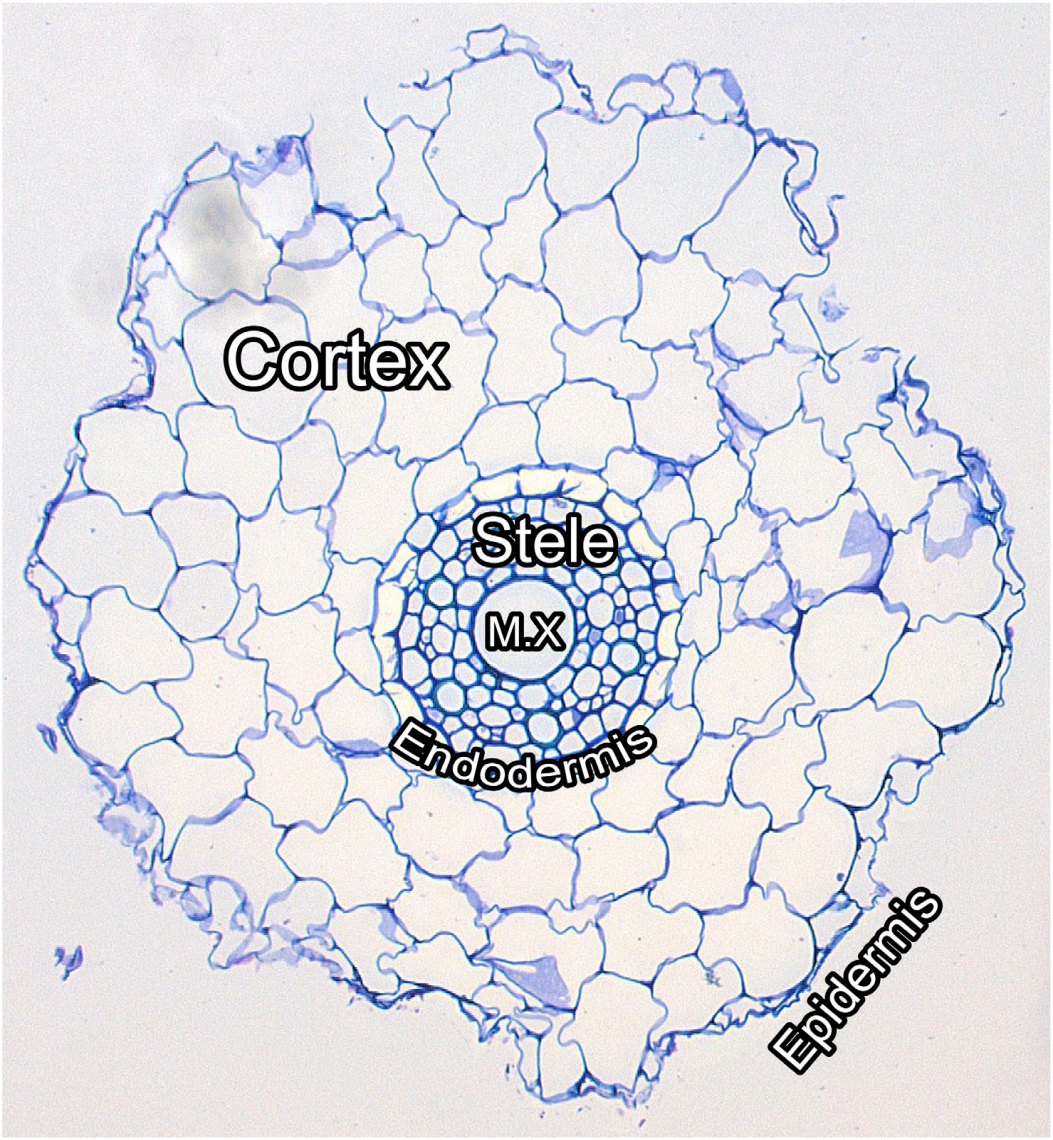
Labelled image of a wheat root cross section. The epidermis is the outermost single layer of cells of the root. The cortex is the second outermost region and houses the largest cells in the root. The endodermis is the single layer of cells forming the boundary between the cortex and the stele. The stele is the set of cells inside the endodermis. Finally, the metaxylem, denoted in the figure by M.X, is located in the centre of the root and is repsonsible for water and nutrient transport.

The difficulty involved in the phenotypic analysis of the anatomy of roots is again the tedious and time consuming manual acquisition of raw data and its subsequent analysis. The usual procedure involves manual slicing of each sample root into lateral or longitudinal sections of very thin width, of the order of 10–100 *μ*m, staining the sections with a dye and then imaging each section with a high resolution microscope-mounted camera, see Fig 1 for an example. Finally, various anatomical traits are measured, manually or semi-automatically, from the microscopic images. The sectioning process is laborious and time consuming but this task is eclipsed by the number of hours required to manually label and extract morphological features, such as area and perimeter, of every cell in every cross sectional image, and provide summary statistics of these features for each tissue region. In this article, we propose a fully automated image-based method for the analysis of lateral root cross sections, alleviating the burden of manual root anatomy phenotyping and enabling high throughput analysis. We refer to this procedure as RootAnalyzer.

Approaches to analysing root anatomy through image processing can be broadly categorized in three ways; manual, semi-automated and automated. Labor intensive manual approaches are not suitable for high throughput analysis. They introduce the risk of subjective assessment and limit the types of features that can be recorded in practice. For example, Haque et al. [26] manually counted the number of cells in each tissue region. On the other hand, Fan et al. [27] and Lipiec et al. [28] manually labelled root tissue regions and approximate tissue areas and perimeters by means of manual polygon fits. One of the most common software packages used to assist in the latter activity is ImageJ [29], a versatile open source software that has been used in many areas of biological image analysis, including root anatomy analysis. ImageJ contains built-in functionality for measuring lengths and areas using simple manually controlled tools [30]. For a semi-automated analysis, the plugin Fiji [31] is commonly used as it comes with many additional tools, allowing users to create their own image analysis pipeline with the desired degree of automation. Inherent to its versatility, Fiji allows for the application of different approaches to the different stages of the analysis. The user, for example, can select to perform image segmentation using a manually chosen global threshold or by using a range of automatic global thresholding techniques such as Otsu’s method [32] or maximum entropy [33]. However, root cross section images often do not have a uniform background and contain varying levels of noise. As such, more advanced segmentation approaches, such as surface fitting [34], may be required. CellSet [35] is a semi-automatic software designed for analysing images of longitudinal root sections from confocal microscopes. MorphographX [36] and Mars-alt [37] are software dedicated to the semi-automated 3D reconstruction and analysis of certain biological organs. None of the above, however, detects and classifies the various cellular structures present in root cross section images.

To the best of our knowledge, the approach in the literature that is closest to full automation is RootScan [20], a software tool dedicated to the analysis of maize and rice root cross section images. RootScan is designed to allow for user interaction at each stage of the segmentation and classification processes. The software utilizes Otsu’s method to automatically set a global threshold and segment the root from the background. Then, the contrast between pixel intensities in the stele and the cortex region is used to detect the boundary of the stele. Aerenchyma and regular cells in the cortex are differentiated by the length of their major and minor axes. Similarly, central metaxylem elements are differentiated from other stele cells by area. At the completion of all of these steps, RootScan saves a table of statistics recorded from the detected cells and root regions. However, some aspects of this algorithm make it unsuitable for the images studied in this article. For example, global thresholding often fails in the presence of noisy and complex backgrounds and when the colour contrast between various tissue regions and cells is not sufficient. Furthermore, there is generally a greater distinction in size and morphology between endodermal cells and cortex cells, in maize than in wheat, making accurate identification of the stele more difficult.

The algorithm proposed in this article takes images of cereal plant root cross-sections as input, segments the root from the image background, extracts and classifies individual cells as well as different tissue regions and finally produces a suite of statistics about those cells and tissues. Our algorithm differs from those available in the literature in four main respects. Firstly, after setting two image resolution-dependent parameters, the approach is fully automatic, which makes it suitable for batch processing of large series of images. Secondly, although originally designed for the analysis of cross-section images of wheat, RootAnalyzer’s scope is not limited to these plants alone. Maize images can also be processed; the user is only required to specify the species. Thirdly, RootAnalyzer is versatile and rarely dependent upon image intensities when classifying regions. Rather, the algorithm uses basic *a-priori* knowledge about root morphology, such as cell and tissue size and locations, which lends the program to applications on a range of species. Lastly, the procedure offers an analysis of root tissues and root cells based on a suite of primary and secondary statistics of root morphology, such as area and eccentricity.

## Materials and Methods

### Plant Materials

The images used in this paper are taken from the roots of two 50–56 day old wheat (*T. aestivum L*) varieties: the Australian cultivar Kukri and the breeding line RAC875. Two different water treatments, i.e., well watered (WW) and drought (Dr), were applied to the two genotypes, with each genotype-treatment combination represented by four biological replicates. The images of root sections were obtained from primary roots at four centimetres from the tip as described by Steinmann et al [24]. The Technovit embedding kit was used to embed the cross sections, which were then sectioned with a Leica VT1200 microtome.

### Image Acquisition

The images of these sections were taken with a Leica AS LMD laser dissection microscope with a DFC 480 camera at 10x zoom. A scale bar was provided with the images so that results of the RootAnalyzer algorithm can be converted from pixels to *μ*m.

### Feature Extraction and Validation

Table 1 summarizes the quantitative features extracted by RootAnalyzer. More detailed secondary statistics have also been recorded in the last stages of the algorithm. This is explained in detail in the Output Statistics subsection. For all features, except eccentricity, we have recorded an associated manual measurement to evaluate the accuracy of the algorithm. Our manual measurements were conducted using Photoshop CS6 by manually selecting vertices of polygons which closely approximate the boundaries of individual cells and regions, or by use of the magic wand tool where appropriate.

**Table 1:** Primary statistical features captured by the RootAnalyzer algorithm. A selection of these is discussed in detail in the Output section.

**Region**	**Feature**	**Description**	**Unit**
Whole Root	Total Area	The area of the entire root section	*μ*m^2^
	Eccentricity	Eccentricity of the entire root section	Eccentricity metric
Tissue Regions (Cortex, Stele, Endodermis, Metaxylem)	Total Area	Area of the tissue region	*μ*m^2^
	Number	Number of cells inside the tissue region	Count
	Cell Area	Average area of cells inside the tissue region	*μ*m^2^
	Cell Eccentricity	Average eccentricity of cells inside the tissue region	Eccentricity metric
Stele	Eccentricity	Eccentricity of the stele region	Eccentricity metric

## RootAnalyzer

**Fig 2:**
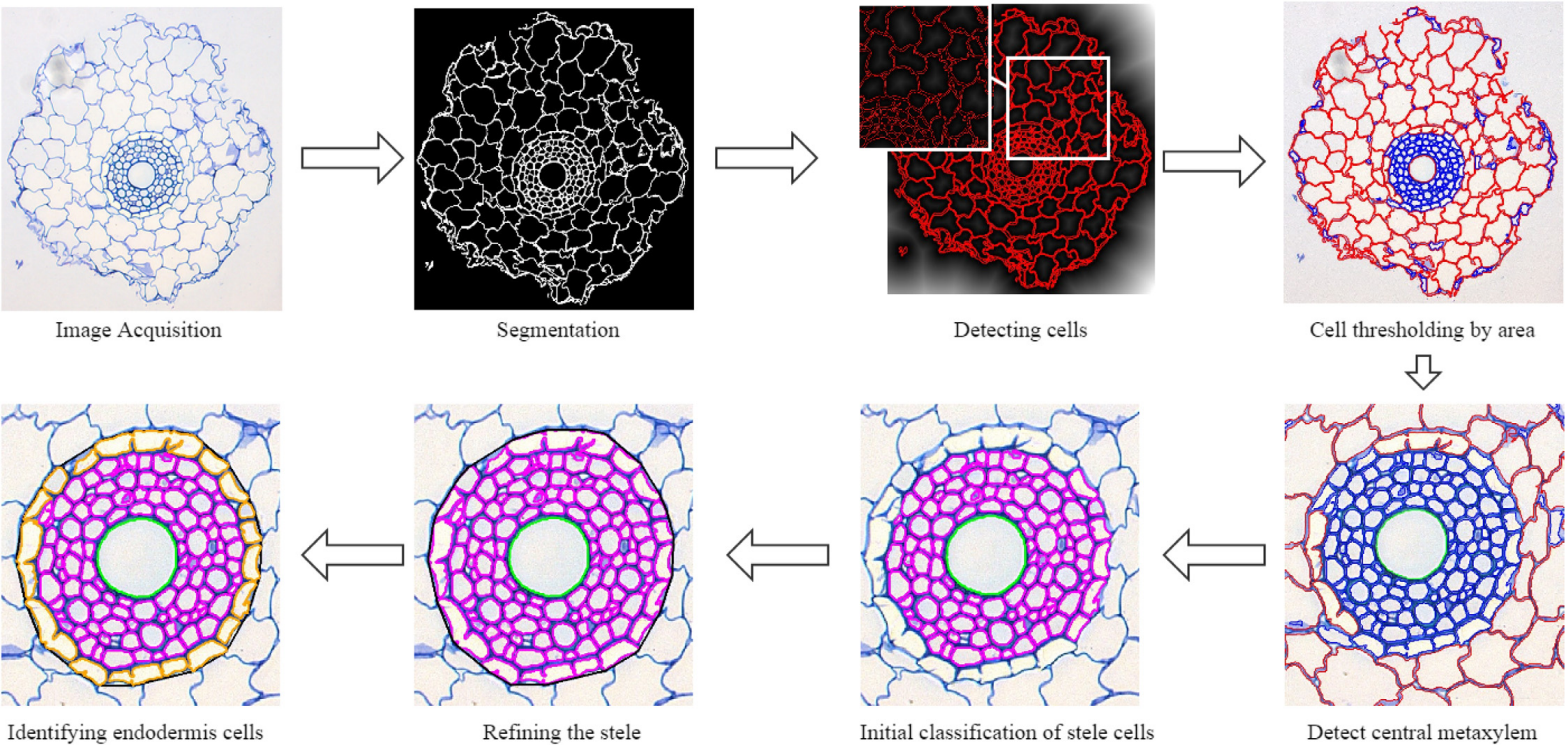
Flowchart outlining the steps of the algorithm. First we acquire the images using a Leica AS LMD laser dissection microscope with a DFC 480 camera and then segment them using a local thresholding technique. Cells are detected using a distance transform of the segmented image and are then initially classified by their area. A number of subsequent steps then correctly classify cells into the four main regions; cortex, stele, endodermis and central metaxylem.

The procedure, called RootAnalyzer, is dedicated to the automatic, efficient and accurate analysis of root cross section images. The program is capable of analyzing not only images of wheat roots but also maize. We also postulate that other species of plants, such as a range of dicots as well as monocots, may be analyzed in an automated manner; we comment on this further in the Results section. The procedure involves a sequence of image processing steps to produce a suite of results without any manual intervention. Fig 2 outlines the different steps of the analysis procedure. As explained in the Image Acquisition section, a camera mounted microscope is used to acquire root cross-section images. The images are first segmented in order to separate the root section, hereinafter called foreground, from background features (Segmentation section). All cells visible in the root cross-section are then automatically detected (see the Root Cell Detection subsection) and classified into tissue types, e.g. stele, cortex, metaxylem, based on size, morphology, shape and spatial distribution (Classifying Cells subsection). Finally, RootAnalyzer produces statistics on a number of features of biological relevance (Output Statistics subsection). We describe each of these steps in the following subsections.

### Segmentation

In the first stage of our algorithm, each image pixel is classified as either belonging to the root or to the background. The most common approach to segmentation is automatic global thresholding [32], which assumes that the pixel intensities follow a bi-modal distribution. Each mode corresponds to either the foreground or background. Global thresholding is particularly suitable for images with uniform backgrounds. In many root images, however, the image regions are not uniform. In addition, the intensity of staining varies throughout the image as well as across the root. To meet these challenges, we have adopted a local thresholding technique. The idea is to analyze the intensities in a small neighborhood around each pixel, rather than all image pixels at once.

Local thresholding computes the difference between the intensity of a pixel, *I*(*x*, *y*), and the local mean intensity of the pixels within a square neighborhood of width 2*w*+1, around the pixel of coordinates (*x*, *y*). This operation produces a difference image, denoted $\hat{I}$, which is expressed as

$$\begin{array}{cc} \hat{I}= & I-(I⋆M), \end{array}$$ (1)

where *M* is the mean filter such that:

$$\begin{array}{cc} (I⋆M)(x,y)= & \frac{1}{{\left(2w+1\right)}^{2}}\sum_{i=x-w}^{i=x+w}\;\sum_{j=y-w}^{j=y+w}I\left(i,j\right). \end{array}$$ (2)

**Fig 3:**
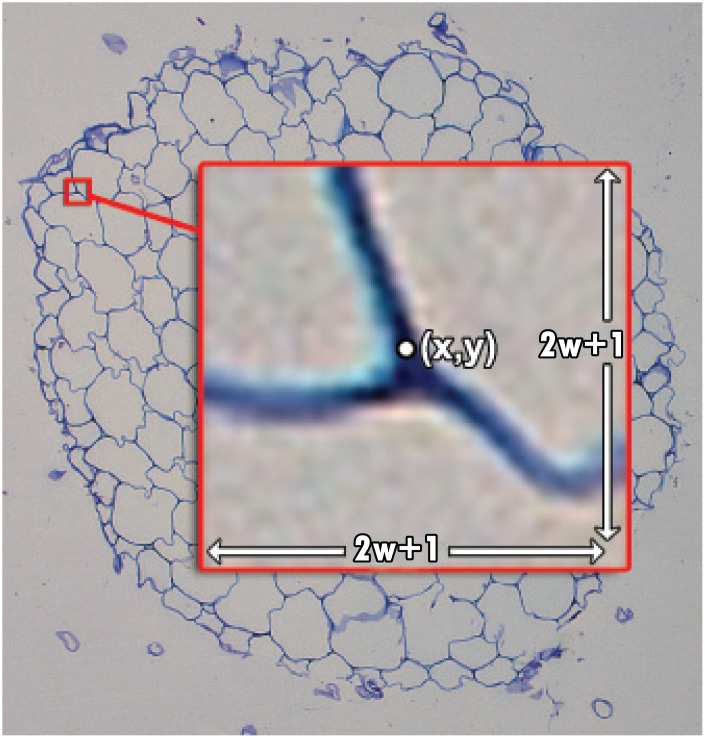
Visual depiction of the local thresholding approach. If the intensity at a pixel (*x*, *y*) differs from the mean intensity inside the window by more than some pre-defined threshold, we conclude that it is a foreground pixel.

Next, a threshold, *T*, is set and applied to the difference image $\hat{I}$. Hence, if $\hat{I}(x,y)>T$ we conclude that *I*(*x*, *y*) is a root pixel. With reference to the illustrative guide in Fig 3, if the pixel at (*x*, *y*) differs by more than *T* from the mean intensity inside the (2*w*+1) × (2*w*+1) window, then (*x*, *y*) is a root pixel. In this paper, we have set *T* = 0.05 and *w* = 12 for all images. We discuss the robustness of these choices in the Discussion section. Note that prior to any analysis, we normalize the image intensities to [0, 1]. This produces a binary image wherein pixels that belong to the root are set to one and others are set to zero.

**Fig 4:**
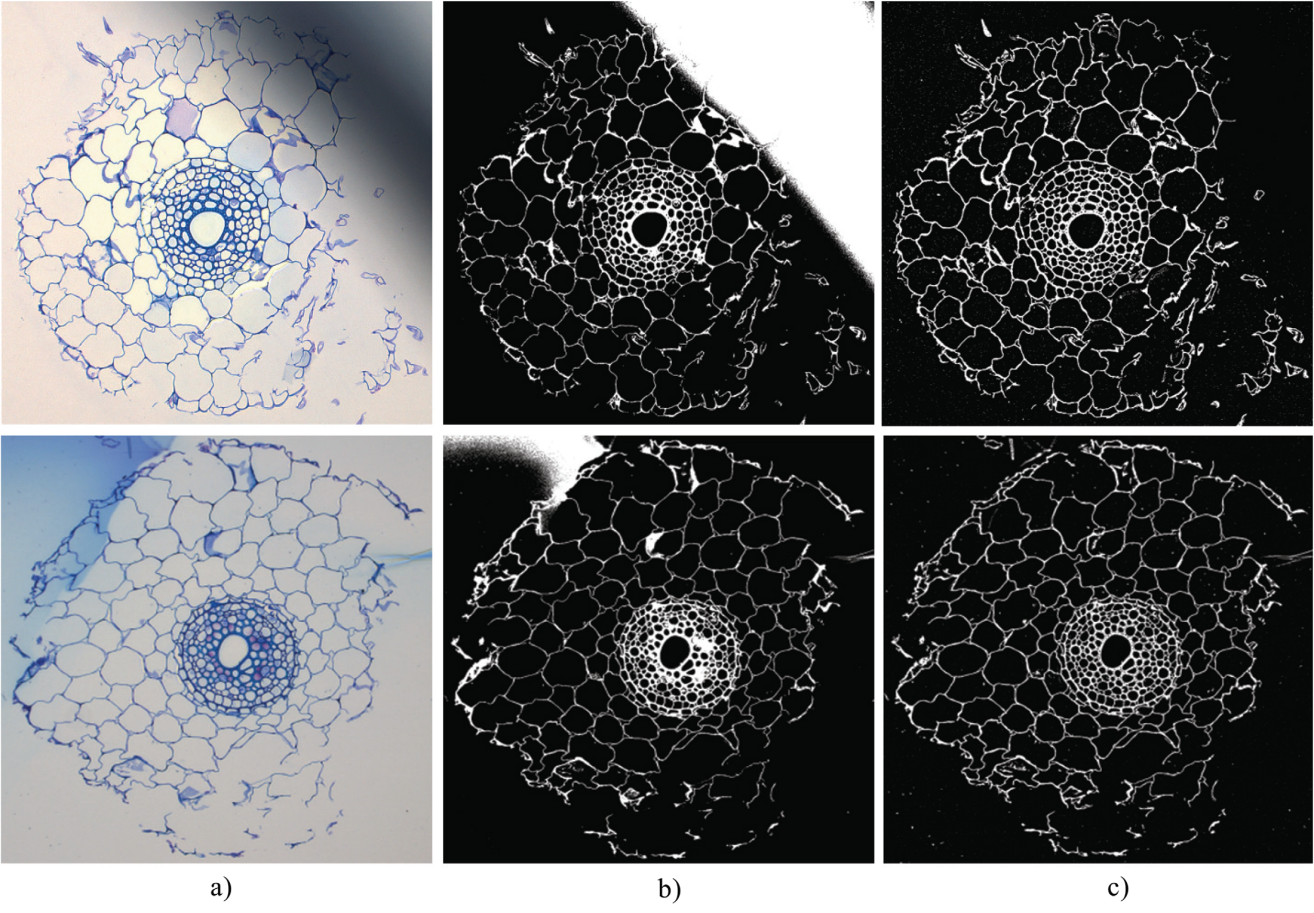
Challenging images make local thresholding a necessity. (a): Input images with regions of varying intensity. (b): Segmentation result using RootScan’s global thresholding method. (c): Segmentation result using RootAnalyzer’s local thresholding method.

In many of the root cross section images we analyzed for this paper, the backgrounds were largely inhomogeneous. Thus, global segmentation often fails. In contrast, the local thresholding method described above was capable of accurately segmenting all images. This is exemplified in Fig 4 where it is demonstrated that large areas of varying intensity, such as the large dark region in the top image of Fig 4(a) and the large blue region in the bottom image of Fig 4a, are correctly dealt with. One artefact of the local thresholding technique, however, is that some small foreground connected components, or noise, are regularly detected. We point out in the Classifying Cells subsection that the area-based cell classification automatically filters out this segmentation noise.

### Root Cell Detection

**Fig 5:**
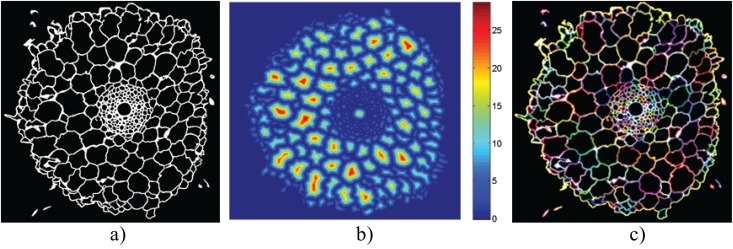
Detecting cells. (a) Binary segmented image. (b) Distance transform applied to the segmented image. Peaks correspond to the centers of cells. (c) Cells are detected by interpolating around each location where the binary image changes from background to foreground.

The segmentation procedure described above produces a binary image *B* (Fig 5(a)) exhibiting foreground pixels corresponding to boundaries of individual root cells. Ideally, the interior regions to these boundaries correspond to the interior of the root cross-section’s cells. In practice, however, some boundaries are incomplete due to imperfect image acquisition or to damage occurring during the root cross-section preparation process itself, see the two examples of Fig 4. To find the exact boundaries of all individual cells we propose a two stage process. The first stage detects the large majority of root cells in an initial segmentation stage. The second stage then searches for any cortex cells that may have been missed and captures them with a subsequent refined segmentation. A detailed description of these two stages follows.

The first stage of root cell detection begins with dilating the binary image *B* with a small 2 × 2 pixel kernel. This step closes over any small leaks in cell boundaries without impacting negatively on the accuracy of small cells such as those found in the stele. The procedure then checks every pixel in the image and identifies those for which *B* changes from background to foreground, *i.e.*, from black to white. Once all such cells have been tagged as belonging to a region’s edge, linear interpolation is performed to iteratively draw a boundary around each cell in the image. At this stage we also detect if any lateral root formations are present and have been mistakenly added as cells. As lateral roots display very low intensities in grayscale images, we remove all regions with a mean intensity that is less than one third of the mean intensity of all detected regions. Finally, the boundary segments of those cells in contact with the background are then concatenated to form an initial estimate of the boundary of the root, Γ_*ini*_.

Poor quality root specimens, poor quality staining or just poor quality images can result in cell boundaries that are either slightly perforated or are too faint to be captured by the local threshold segmentation process. This results in a segmented image with some incomplete cell boundaries. The interior of these cells can then be mistakenly considered as belonging to the background region. To correct for this we enter a second segmentation stage wherein the problem is overcome by halving the local threshold, *T*, which results in a segmentation that is far too noisy for regular cell detection, but that often completes open boundaries of affected cells. At this stage, we calculate the Euclidean distance transform of the binary image *B*. A new image, *D*, is thus created (Fig 5(b)) wherein each pixel stores the Euclidean distance to its nearest non-zero (foreground) pixel in *B*. The peaks of this distance map *D* indicate where cell centres should be located. Hence, if a peak is located outside of the root’s initial boundary estimate, Γ_*ini*_, but inside a newly identified cell interior, then that cell is added to the set of already acquired root cells. The root boundary is then recalculated (Γ_*final*_). The noise created through this (temporary) refined segmentation step is discarded. The final result of individual cell detection is illustrated in Fig 5(c).

### Classifying Cells

While many existing approaches [20, 29] to classifying cells into different regions such as the stele and cortex use contrasting pixel intensities, we have chosen to create a multi-stage process assuming some basic a-priori biological knowledge of root tissue structure. First, we assume that epidermal cells are, on average, smaller than cortex cells. Second, we assume that, on average, cells inside the stele and endodermis will also have a smaller area than cells in the cortex. Hence, area-based thresholding of cells into ‘small’ and ‘large’ categories, neglecting epidermal cells for the moment, leads to a cluster of small cells that approximates the union of stele and endodermis regions.

Based on considerable physiological evidence [38], the next assumption we make is that one or more metaxylem cells will reside near or about the centre of mass of this small cell cluster. We therefore classify any relatively large cell that is completely surrounded by small cells in this clustered region, as metaxylem, provided that its area is significantly greater than the mean area of all these cells. The centre of mass of the metaxylem cells is then used as an approximation to the centre of the stele. This allows us grounds to further classify cells in the stele and other tissues. Below, we describe these steps in detail.

#### 1) Detecting epidermal cells

Epidermal cells making up the outer root boundary are generally challenging to capture, as they may vary in size and are occasionally completely absent in places due to imperfect image quality or damage of root samples. When absent, cortical cells become those that falsely form the root perimeter in an image. To only capture true epidermal cells, RootAnalyzer examines the distribution of area of the cells making up the root’s perimeter. By assuming that the majority are actual epidermal cells while only a few captured cells are cortex cells, it remains only to find a suitable threshold that will remove cortical cells from the list of candidate epidermal cells. Here we present three potential approaches to achieve this, their advantages and disadvantages and which of the approaches proved most robust.

The first approach involves smoothing the distribution of cell areas and subsequently detecting the first local minimum in the smoothed distribution. When the epidermal cells are approximately of a constant size this generates a distinctive local minimum between the epidermal and cortex cell areas. The approach can, however, fail when the epidermal cell area is highly variable, creating local minima within the epidermal cell area distribution itself. A second approach is similar, but rather than searching for the first local minimum, cell areas are binned in a frequency histogram. The first empty bin is sought and its location is used as the area threshold. This method is useful for good quality roots with approximately uniformly sized epidermal cells, but is prone to errors arising from poor choice of bin size. The third approach, which we have chosen to utilize in RootAnalyzer, sets the threshold based on a specified percentile of the data. For the root images studied in this article we found that epidermal cells make up roughly 80% of the cells contributing to the root boundary. By sorting candidate cells according to increasing area, we classify the first 80% of candidates as epidermis cells, the remainder as cortex cells.

The third, percentile approach produced the best result of the three methods outlined here, as it allowed for some variation in epidermal cell area commonly present in most roots. However, the second, histogram approach gave comparable results. A potential problem with the percentile approach, would arise in the case of a high quality root image whose perimeter is made up almost entirely of epidermal cells. In this ideal case, approximately 20% of the epidermal cells would be misclassified as cortex cells. In this case, the histogram approach would perform better. However, even the highest quality images analyzed in this paper still exhibit sufficient variability in epidermal cell size to warrant use of the percentile approach.

#### 2) Cell thresholding by area

**Fig 6:**
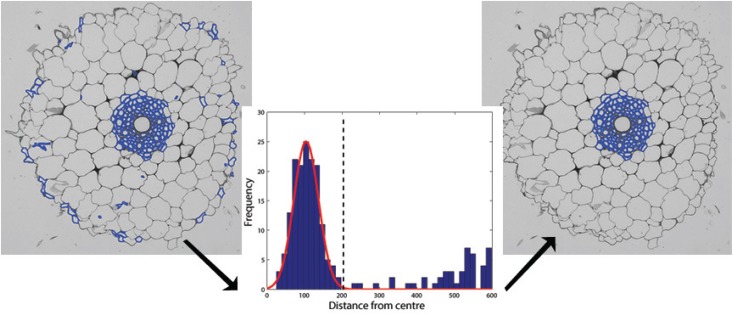
Finding the central metaxylem. Left: Collection of all small cells. Middle: Histogram showing the distance from the centre of each cell to the centre of the root vs frequency. The peak at low distances corresponds to cells inside the stele and endodermis. Right: Keeping cells whose distance is greater than the illustrated threshold provides a first estimate of stele and endodermis cells.

We assume that cells inside the stele and endodermis are significantly smaller than cells in the cortex. Thus, we first categorize every non-epidermal cell as either small or large, based on an area threshold. The value of this threshold will depend on image resolution and should be assigned a-priori. For the images shown and analyzed in this paper, the two thresholds *A*_*s*_, minimum area of small cells, and *A*_*l*_, minimum area of large cells, are set to 50 square pixels and 1000 square pixels, respectively. While the magnitude of these thresholds will change with root size, we show in the Discussion section that their ratio is quite robust. While area-based thresholding provides a good initial classification, this criteria alone is not sufficient to unequivocally determine stele and endodermis cells since many small cells are also scattered throughout the cortex, see Fig 6.

#### 3) Finding the metaxylem

We assume that the centre of mass of the small cells is the centre of the stele. Taking a histogram of distances between this approximate centre and the centres of all small cells yields a distribution exhibiting a peak at small distances, see Fig 6. Fitting a Gaussian to this distribution and applying a threshold at +3 standard deviations provides a first approximation to identifying those cells inside the stele. As stated above, this simple approach does not accurately classify all stele cells. However, in all cases that we have tested, the metaxylem have been successfully captured inside this initial boundary. Finally, we assume that metaxylem cells will be significantly larger than other stele cells and that all of their closest neighbors will either be other metaxylem cells or other stele cells.

#### 4) Classifying stele cells

**Fig 7:**
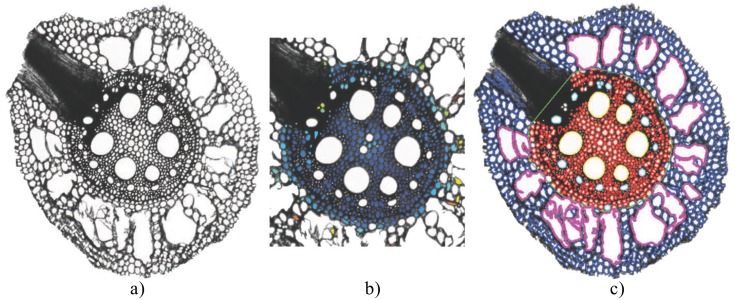
Sample result of RootAnalyzer applied to a maize image. (a) The original maize image. (b) Illustration of the classification process. Deep blue represents a higher likelihood of belonging to the stele or endodermis. (c) The result after all steps of the algorithm are complete. Blue cells belong to the cortex, magenta cells are aerenchyma, red cells belong to the stele and endodermis, cyan cells are protoxylem elements and yellow cells are central metaxylem.

We use the following approach to classify cells belonging to the stele and endodermis and thus determine their tissue boundaries. First, we compute the pairwise distances between cells that are considered close to the metaxylem. The underlying assumption is that cells inside the stele, or on the stele boundary, are mostly surrounded by other stele cells. Cortex cells that have been misclassified as endodermis cells would exhibit large average distances to other small cells. By considering a small number of nearest neighbours, the maximum distance from a given cell to any of its neighbour cells can be used as a measure of likelihood that the cell is inside the endodermis or stele. This is illustrated in Fig 7(b) by the deep blue coloured cells having small distances and yellow/orange coloured cells having large distances. In this paper, we have considered five nearest neighbours and set a distance threshold of 50 pixels.

#### 5) Refining the classification of stele cells

In practice, the above classification method can sometimes fail to capture a few stele or endodermis cells. Two factors are responsible for this deficiency. Firstly, cells that have been categorized as large in area, that are not metaxylem cells, such as protoxylem elements, should be classified as belonging to the stele or endodermis. A simple check that every point on such a cell’s perimeter resides inside the general boundary of the endodermis is sufficient to resolve this ambiguity. The second issue is the misclassification as cortex cells of those stele or endodermis cells that lie just beyond the approximate boundary of the endodermis region. To solve this problem, we determine if the small and large cells satisfy the joint condition that they have an eccentricity value above 0.9 and are within ten pixels of the approximate endodermis boundary. If these conditions are satisfied, we conclude that they belong to the stele or endodermis. The majority of these cells actually belong to the endodermis and will be correctly classified in the next stage of the algorithm.

The final step in refining the stele and endodermis regions involves removing false positives, *i.e.*, cortex cells that have been classified as belonging to the endodermis. To do so we check the nearest neighbouring cells to every point of a cell’s boundary. If more than 50% of the neighbours are from the cortex then we conclude that the cell in question must also be from the cortex.

#### 6) Identifying endodermis cells

Once all cells have been correctly classified as belonging to either the stele or the endodermis, the last step is to specify to which of the two regions a cell actually belongs. To this end we simply assign to those cells closest to the boundary of this cell cluster, the role of endodermal cells.

This concludes the collection of regions that are identified by the algorithm. The improvement gained after implementing these steps can be seen in the bottom row of Fig 2.

#### Implementation

The RootAnalyzer algorithm was implemented in MATLAB 2013a and converted into an executable file. Thus, it can be run on any machine without any version of MATLAB installed. The program operates in a command-line mode. The operational parameters can be specified in an associated text file. A user guide, downloadable along with the executable, describes how to correctly define parameters in this text document. The user guide also provides tips on how to select optimal parameters.

### Extension to Maize Images

The procedure described above is loosely based on the assumption that the root contains a single, central metaxylem cell. However, as we discuss later in this article, the procedure also applies to roots possessing multiple metaxylem cells provided they are distributed approximately symmetrically around the stele centre, for which there is some anatomical evidence [38]. Nevertheless, we have described the above algorithm based on our primary interest of analyzing cross-section images of wheat roots. However, only a slight modification to the pipeline is required to analyze images of maize. The extended algorithm includes the added feature of protoxylem and aerenchyma detection.

#### Protoxylem and aerenchyma detection

In wheat, protoxylem cells are often indistinguishable from xylem parenchyma cells and aerenchyma are not present. Hence, detecting these features in maize images requires additional steps. However, this is straightforward using our initial classification of large and small cells based on area. We classify all large cells inside the stele that aren’t metaxylem as potential protoxylem elements. The last step of protoxylem detection is to calculate, for each protoxylem candidate, the line that begins at the stele centre, passes through the protoxylem element and ends at the stele boundary. All candidate cells located more than half way along their respective lines are classified as protoxylem elements. For the aerenchyma cells, we approximate the distribution of large cells inside the cortex by two Gaussians with the larger of the two mean sizes being the aerenchyma cells and the smaller being regular cortex cells. It remains to set a threshold between the two distributions such that all cells with area larger than the threshold will be classified as aerenchyma. As the aerenchyma cells are substantially larger in area than regular cortex cells, this threshold can safely be set at one standard deviation less than the mean of the distribution of larger cells. Fig 7 shows an example application of this extended procedure to a sample maize image.

### Output Statistics

Table 1 summarizes the features quantified by RootAnalyzer. We partition these into primary and secondary statistical data.

#### Primary data

The predominant features are geometric: the area of each region and the number, area and eccentricity of cells in each region. Additionally, the eccentricity of the whole root and stele region are calculated. The eccentricity *E* of an object is determined by the formula $E^{2}=1-\frac{b^{2}}{a^{2}}$, where *a* is the length of the major axis and *b* is the length of the minor axis of an ellipse that has been fitted to the object’s boundary by least squares optimization. Often in our images the plant root only contains one central metaxylem. In such cases, the average area and eccentricity statistics refer to data for a single cell rather than an average over multiple cell values.

Epidermal cells are prone to damage during the cutting procedure and are often split into a number of cell fragments. Manual ground truth labelling is then often inaccurate. For this reason and although we detect the root’s epidermal cells as discussed, we do not report on the accuracy of their characterization.

#### Secondary Data

**Fig 8:**
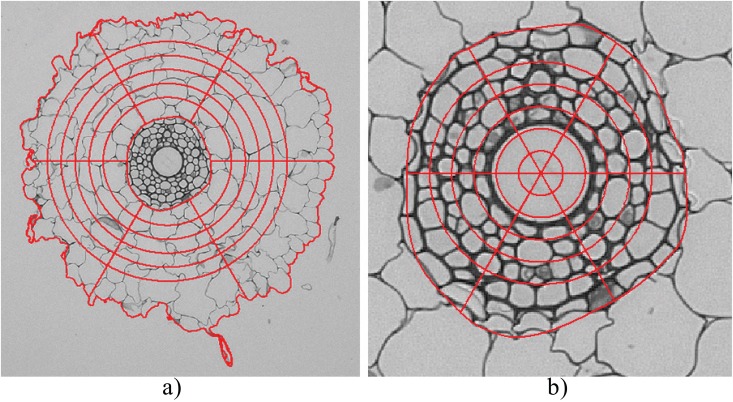
Sample of the cheesewheel approach. (a) The entire root section with partitioned cortex. (b) Cheesewheel partition of the stele and endodermis. In (a) the inner boundary of the inner-most annulus is created using the boundary of the endodermis as a guide. Similarly, the outer boundary of the outermost annular region is created using the boundary of the epidermis as a guide.

The secondary statistical features quantified by the algorithm are illustrated visually in Fig 8. Overlaid on the stele, endodermis and cortex regions, are six annuli of approximately equal radial width. These are further divided into six wedges of equal length. By averaging primary statistics over each of the six wedge regions for a given radius we obtain meaningful statistics that uncover trends as a function of radius from the root centre. For instance, within the stele, the largest and most distant annulus displays higher average cell eccentricity than do inner annuli, indicating that this region houses endodermal cells.

## Results

The RootAnalyzer procedure has been applied to the dataset outlined in the Materials and Methods. The dataset contains 15 images consisting of 4 well-watered and 4 drought stressed Kukri root cross-sections, 4 well-watered RAC875 cross sections and 3 drought stressed RAC875 cross-sections. After applying the algorithm to these images, the results are compared to manually obtained ground truth values, as discussed in the Materials and Methods. The error in feature characterisation and visual illustrations of the results are given in the following subsections.

### Segmentation

We have successfully applied the local thresholding approach used in this work to the 15 wheat images. In contrast, an automatic global threshold often delivered poor segmentation results for our images, preventing successful application of the algorithm. Fig 4 shows examples of the significant difference in the quality of segmentation using the two segmentation approaches. Although segmentation by local thresholding is slower than by global thresholding, it still only takes on average 2.5 seconds per image of resolution of 1024 × 1024 pixels.

### Cell Classification

**Fig 9:**
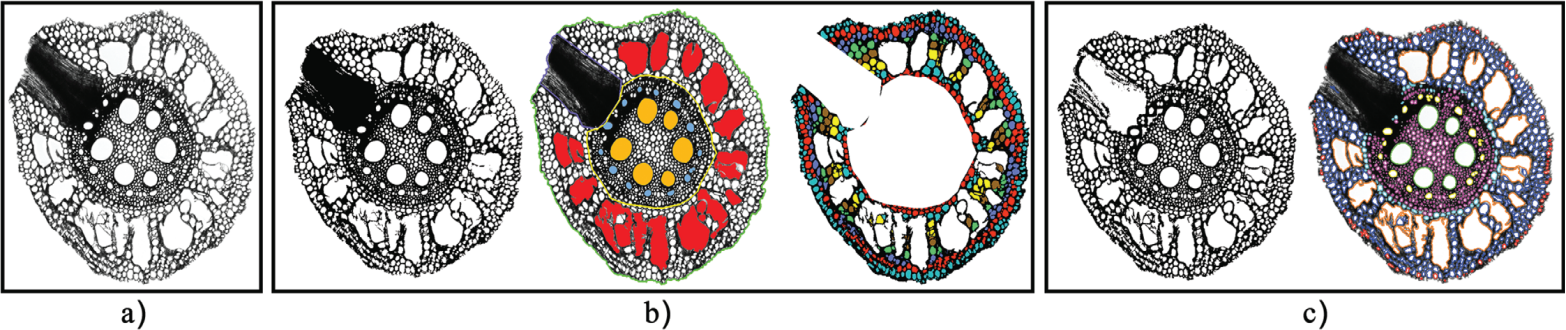
RootScan and RootAnalyzer applied to a sample maize image. (a) The original image. (b) Results from RootScan. (c) Results of RootAnalyzer.

Fig 9 shows two sample results of the cell classification of the RootAnalyzer algorithm. Note that all cortical cells and the challenging stele cells have been properly captured. The endodermis and central metaxylem have also been correctly identified in each case. An infrequently occurring issue, illustrated in the bottom row of Fig 9(a) by a magenta rectangle, is the segregation of some part of the root. The problem of segregated regions is difficult to overcome since any significant noise in the background can easily be confused with isolated root regions. However, such problems arise infrequently. Also, the impact of such errors can potentially be minimized by averaging data acquired over many samples in a given experiment. We illustrate this effect on our relatively small data set in the Statistics section.

#### Comparison with RootScan

**Fig 10:**
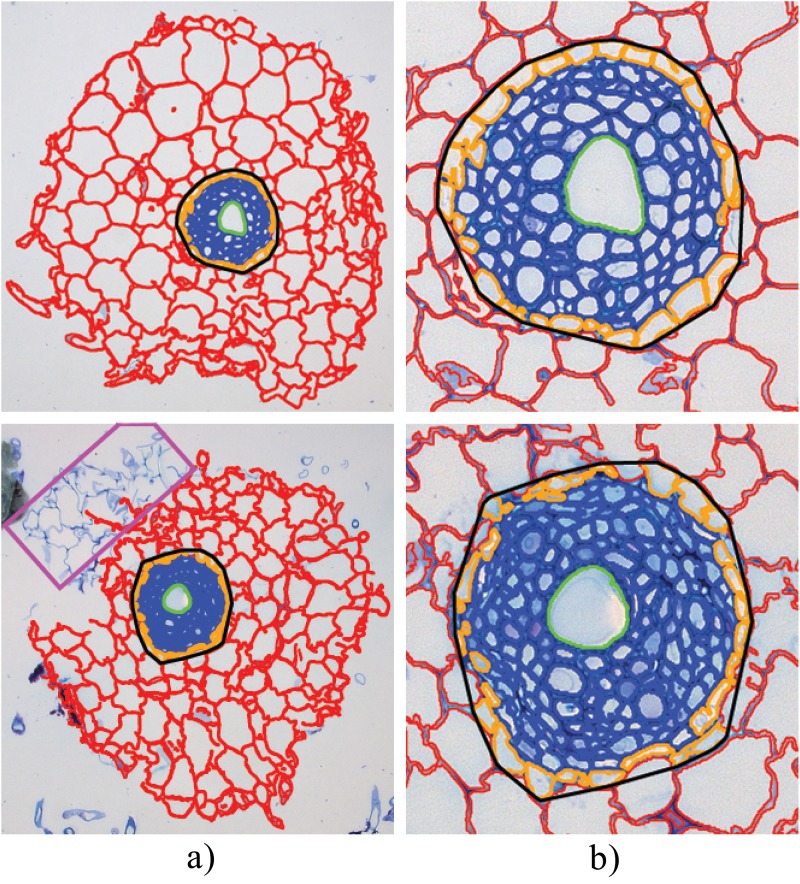
Sample results from the RootAnalyzer algorithm. (a) the entire root section analysed. The magenta rectangle in the bottom image denotes a small piece of segregated root that the algorithm has failed to capture. (b) zoomed versions of the respective stele regions highlighting cortical cells (red), epidermal cells (orange), stele cells (blue) and central metaxylems (green).

Fig 10 illustrates the result of application of both RootScan and RootAnalyzer to a typical maize image. Note, however, that RootScan was strictly developed for application to maize and rice images but not wheat. Fig 10(a) shows the original image. In Fig 10(b) we show the results of the RootScan [20] algorithm applied to the maize image in an automated manner. RootScan is by design a semi-automated software and performs very well when operated this way. With a few clicks of the mouse in missed regions, or through dragging some boundary points, RootScan is capable of producing results nearly indistinguishable from the ground truth. RootAnalyzer, however, is designed as an automated software. To demonstrate the usefulness of RootAnalyzer, for example in situations where manual intervention is impractical, we have chosen to present RootScan’s results following an automated analysis as a comparison. Note that RootScan requires lateral root formations to be removed manually and without doing so the algorithm fails. All other stages of the algorithm were run without user intervention when obtaining the following results.

The left-hand panel in Fig 10(b) shows the original image after segmentation by RootScan. The middle panel shows the different regions, classified by colour. On the right, cortex cells have been partitioned by their radial distance from the stele boundary. Fig 10(c) shows the successful segmentation (left) and classification (right) of cells of maize, by the RootAnalyzer algorithm proposed in this paper. The results visualized in Fig 10 have been quantified in Table 2, where the empty entries correspond to statistics captured by RootAnalyzer, but not by RootScan. Table 2 demonstrates that the two algorithms excel in different areas. RootAnalyzer produces 10.9% and 10.1% error in recording the number and area of cortex cells, respectively, whereas RootScan produces 27.3% and 34.4% error for the same statistics. Alternatively, RootScan finds the exact number of protoxylem elements and only produces 10.1% error in recording their area. RootAnalyzer overestimates the number of protoxylem elements by four, however their area is still recorded with an error of only 12.4%.

**Table 2:** Primary statistics captured by the RootAnalyzer algorithm when applied to the example maize image. The error of each measurement, as compared to the manually obtained ground truth, is also included.

**Region**	**Feature**	**Ground Truth**	**RootAnalyzer (error)**	**RootScan (error)**
Root	Area	447,750	424,398 (5.2%)	454,158 (1.5%)
Cortex	Number	644	714 (10.9%)	468 (27.3%)
	Cell Area	100.1	110.2 (10.1%)	134.4 (34.3%)
Stele	Area	141,446	134,495 (4.9%)	116,138 (17.9%)
	Number	724	673 (7.0%)	-(%)
	Cell Area	20.9	22.3 (6.7%)	-(%)
Endodermis	Number	56	66 (17.8%)	-(%)
	Cell Area	100.5	71.5 (28.9%)	-(%)
Metaxylem	Area	2,687	2,668 (0.7%)	3,447 (28.28%)
	Number	6	6 (0%)	4 (33.3%)
Protoxylem	Area	252.6	221.1 (12.4%)	278.2(10.1%)
	Number	13	17 (30%)	13 (0%)
Aerenchyma	Area	4,467	4,170 (6.7%)	4,856 (8.7%)
	Number	16	17 (6.3%)	17 (6.3%)

### Statistics

Table 3 provides an overview of the statistical information produced by the algorithm and associated errors as compared to manually measured quantities. In this article we have studied 15 images of wheat root cross sections. The images exhibited varying image quality, root specimen qualtiy and quality of staining. While the degree of quality in these aspects varies over the set of images, 13 of which were deemed as acceptable overall quality. RootAnalyzer quantified the features of interest for all regions to an accuracy of approximately 90% or better, averaged over all acceptable images. In particular, the average error recorded for statistics regarding the number and area of cells in the stele and cortex regions never exceeded 7%.

**Table 3:** Primary statistical averages and errors thereof of features captured by the RootAnalyzer algorithm; averages are over all 15 wheat images (columns 3–5). In columns 6 and 7, the degrees of error result from using a subset of images only: 13 high quality images and 2 poor quality images, respectively.

		**Avg. measurement**		**Avg. error (%)**		
Region	Feature	Manual	Algorithm	Overall	Good root sections	Bad root sections
Root	Area	485,156	451,240	6.9%	11.3%	6.2%
Cortex	Number	113.4	103.3	10.1%	6.8%	31.4%
	Cell Area	2,673	2,957	11.2%	6.44%	42.0%
Stele	Area	48,493	49,551	5.9%	4.9%	12.0%
	Number	158	153.7	4.3%	4.3%	4.0%
	Cell Area	133	129.6	5.2%	5.6%	2.6%
Endodermis	Number	26	26	4.0%	4.5%	0.0%
	Cell Area	237	230.9	10.5%	9.6%	16.5%
Metaxylem	Area	3,939	3,907	2.1%	1.9%	3.4%
	Number	1.07	1.07	0%	0%	0%

**Fig 11:**
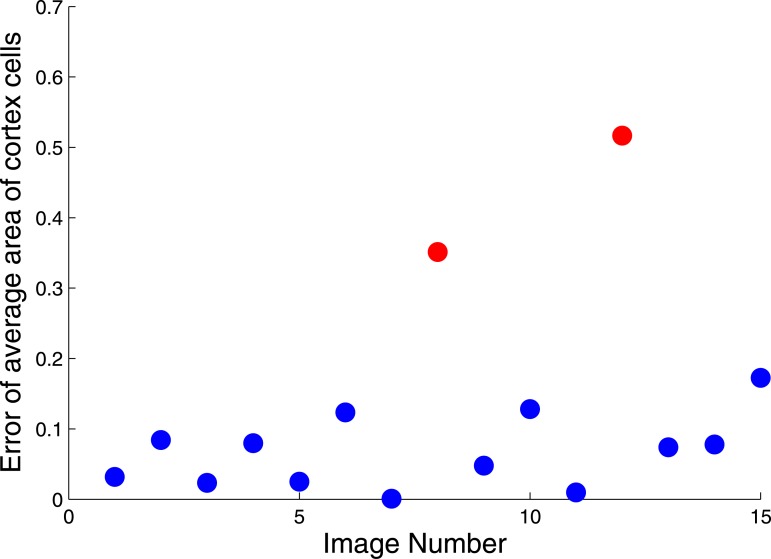
Two images from the set of 15 showing damaged roots. The two images depict damaged root cross-sections with missing or displaced epidermis and cortex regions. The damaged sections are major factors contributing to the relatively large errors in cortex (and presumably epidermis) cell calculations.

**Fig 12:**
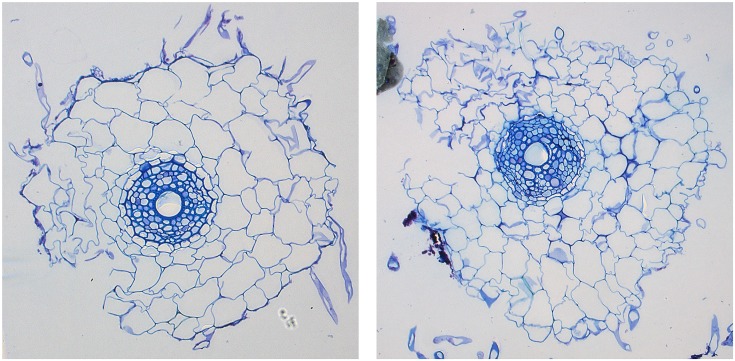
Percentage errors in evaluating average area of cortex cells. 13 of the 15 images produce a much lower degree of error in average cortex cell area calculation. The two red markers of significant error correspond to poor quality (*i.e.*, damaged) root images.

Naturally, the degree of accuracy possible in an application will be dependent on the quality of an image as well as on the condition of the root section itself. While 13 images from the data set are of an acceptable quality, the two remaining images exhibit cortex regions that are particularly damaged or poorly stained. The two root images in question are depicted in Fig 11 and are responsible for the two red points on the scatterplot in Fig 12 representing significant errors compared to manual measurements of the cortex. In each case, a significant fraction of the root (left side of the root) appears damaged; in some cases the damaged section can be partially or wholly dislodged from the rest of the root. While the features of interest for all other regions maintain an acceptable level of accuracy, the number and area of cortex cells in these images produce an error of 31.4% and 42.0% when compared to the ground truth.

The overall accuracy of the algorithm, when averaging over images of both acceptable and unacceptable quality, remains at approximately 90% or better for each feature from each region. The tissue region with the best overall accuracy are the metaxylem cells. As these cells are easily distinguishable when compared to the surrounding stele cells, and generally have clearly defined boundaries, this result was expected. The worst error recorded was only 11.3% and corresponded to the area of the entire root. The cause of this error is primarily broken cells and gaps in the root boundary that were too large to be amended in the epidermal detection stage.

## Discussion

In order to be a useful tool for plant biologists, an algorithm such as RootAnalyzer must meet a number of requirements. First, the tool should be accessible, easy and intuitive to use for the non-expert. Second, given the possibility of a large amount of data to analyze, the tool must be automated while maintaining a high level of accuracy and reasonable speed. Automation for a particular plant species, as primarily demonstrated in this paper, is important. However, to have greater impact the algorithm should be versatile enough to treat a variety of plant species.

### Accuracy

The accuracy of RootAnalyzer is displayed in Table 3. The quantitative results derived by the algorithm are better than 90% accurate, generally, when compared with manually obtained ground truth. Apart from cases of compromised image quality, other instances exhibiting larger percentage errors can equally well be attributed to inaccurate manual measurements. This is exemplified in Fig 12, where cortex cells near the epidermis are small and intricate, making manual measurements challenging. In such cases RootAnalyzer offers another advantage over manual data analysis. Not only is the algorithm automatic and hence less time consuming than manual analysis, it may also be less subjective and more accurate in certain tissue regions.

### Automation

The local thresholding approach that we have employed proved robust to large variations in staining intensity and contrast and high levels of noise. All images analysed in this paper, including the example maize image, were segmented without user intervention. The classification of root tissue regions was also automatic for all images, after initial selection of RootAnalyzer’s input parameters. Once selected, the same parameter values were applicable to all images studied, allowing for a fully automated analysis of a large series of like images.

RootAnalyzer’s parameters are, however, resolution dependent and a new series of images may require adjustment of parameters. To analyze the software’s sensitivity to these parameters, we have simulated a varying resolution of our images by increasing and decreasing their size and investigated the resultant ranges of acceptable parameter values. Our analysis shows that for images zoomed or shrunk to at most double or half their original size, respectively, all parameters defined in the RootAnalyzer section of this article, apart from two, could be kept constant. Furthermore, we found that the two variable parameters in question, *A*_*s*_ and *A*_*l*_, the areas defining the minimum sizes of small and large cells, could be reduced to a single parameter. In all cases studied, we found that choosing *A*_*l*_ = 33*A*_*s*_ provided an adequate initial approximation of stele and cortex cells. Finally, by selecting suitable values for *A*_*l*_ over incremental changes in image size, we have seen that this parameter roughly exhibits a quadratic relationship with the diameter of the root cross section. Specifically, denoting by *d* the cross sectional diameter of the whole root, an estimate for *A*_*l*_ can be obtained by using the equation *A*_*l*_ = 0.0004*d* ^2^+0.668*d* − 112.

As previously stated, the definition of an automated procedure for phenotypic analysis includes the ability to accommodate some degree of variation in the data being analyzed. RootAnalyzer has been formulated in such a way that many of the assumptions made to assist cell classification hold for multiple species of plants. For example, one such assumption is that the centre of mass of metaxylem cells is close to the centre of the stele. This assumption clearly holds for wheat images, given that the tips of primary roots of wheat contain only one central metaxylem, which is centred approximately at the centre of the stele (see Fig 9). Despite the tissue regions of maize, exemplified by Fig 10, having considerably different morphology to those of the wheat root, the same assumption holds. That is, as the multiple metaxlyem of the maize root are approximately distributed symmetrically about the stele in a polyarch arrangement, the centre of mass is again a good approximation to the stele centre. Furthermore, while aerenchyma are only present in the maize image studied in this paper, they can also form in adventitious roots of waterlogged wheat plants [39]. In this case, the extended procedures that have been made for RootAnalyzer are applicable to those wheat images.

While this article shows successful results for wheat and maize roots, which are both monocots, we postulate that segmentation and classification of dicot species will also be possible. Due to the symmetric polyarch arrangement of metaxylem cells in dicots, the centre of mass assumption will generally apply. Furthermore, the distinction in area between metaxylem, stele and cortex cells appears to be similar to that of monocot species.

One potential challenge may be inconsistencies or variations in the staining process. For example, different colours and consequently different grayscale intensities marking different cells. This might be resolved by classifying pixels based on thresholds estimated from different RGB channels.

### Computation time

The average processing time of the RootAnalyzer algorithm was approximately 35 seconds per image, using a machine with an Intel Core i7-2720QM CPU @220GHz processor and 8 gigabytes of RAM. In contrast, RootScan took approximately 80 seconds per image when manual intervention was not required. As a comparison, manual measurements of the same images took approximately 30 minutes per image.

## Conclusions

In conclusion, RootAnalyzer is a fully automated, accurate approach to the phenotypic analysis of root cross sections of primarily cereal plants, with the potential application to many different species. The algorithm required no user intervention for any of the images analyzed, including the challenging stages of segmentation of plant foreground from a noisy background and reconstruction of damaged cellular structures. We have shown that RootAnalyzer exceeds 90% accuracy in quantifying most tissue properties. The algorithm has been formulated in such a way that classification of tissue regions is rarely dependent upon image intensity but rather on morphological properties consistent with a given, if not many, plant species. This invites, as future work, application of the algorithm to different plant species, both monocots and dicots. Another direction for future work is the characterization of more intricate, challenging tissues and regions such as the Casparian strip or cell wall thicknesses.
