# Correction: RootAnalyzer: A Cross-Section Image Analysis Tool for Automated Characterization of Root Cells and Tissues

**DOI:** 10.1371/journal.pone.0143270

**Published:** 2015-11-13

**Authors:** The PLOS ONE Staff

There are errors in Figs 9–12. In Figs 9 and 10, the images and captions have been switched. In Figs 11 and 12, the images only have been switched. Please view the correct Figs 9–12 and their captions here. The publisher apologizes for the errors.

**Fig 9:**
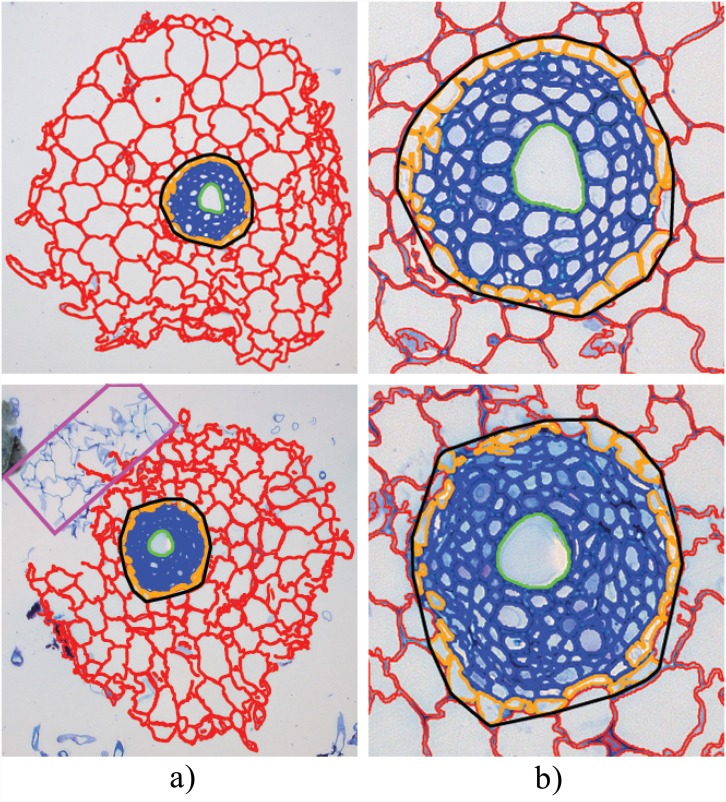
Sample results from the RootAnalyzer algorithm. (a) the entire root section analysed. The magenta rectangle in the bottom image denotes a small piece of segregated root that the algorithm has failed to capture. (b) zoomed versions of the respective stele regions highlighting cortical cells (red), epidermal cells (orange), stele cells (blue) and central metaxylems (green).

**Fig 10:**
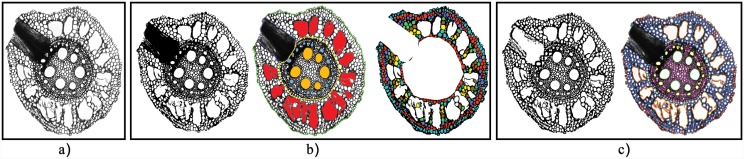
RootScan and RootAnalyzer applied to a sample maize image. (a) The original image. (b) Results from RootScan. (c) Results of RootAnalyzer.

**Fig 11:**
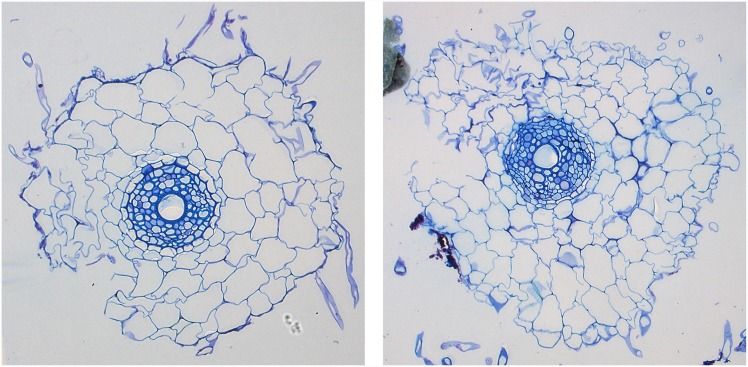
Two images from the set of 15 showing damaged roots. The two images depict damaged root cross-sections with missing or displaced epidermis and cortex regions. The damaged sections are major factors contributing to the relatively large errors in cortex (and presumably epidermis) cell calculations.

**Fig 12:**
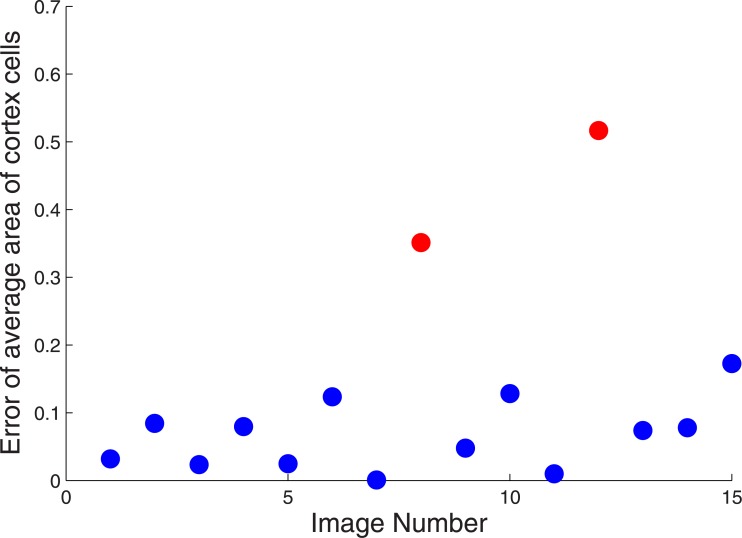
Percentage errors in evaluating average area of cortex cells. 13 of the 15 images produce a much lower degree of error in average cortex cell area calculation. The two red markers of significant error correspond to poor quality (i.e., damaged) root images.
